# Beyond Eosinophilia: A Dual Eosinophil Phenotype Model in Bronchiectasis

**DOI:** 10.3390/medicina62101982

**Published:** 2026-10-14

**Authors:** Hasret Gizem Kurt, Ayperi Öztürk, Zeynep Erayman Özen, Emire Pınar Seyfettin Çelik, Mukaddes Şimşek

**Affiliations:** 1 Department of Chest Diseases, Ankara Medipol University Faculty of Medicine, Özel Bilgi Hospital, Ankara 06000, Türkiye; 2 Department of Chest Diseases, Interventional Pulmonology, University of Health Sciences, Ankara Atatürk Sanatorium Training and Research Hospital, Ankara 06200, Türkiye; drayperi@yahoo.com (A.Ö.); zeynep_erayman@yahoo.com (Z.E.Ö.); pinarseyfettin@yahoo.com.tr (E.P.S.Ç.); dr.mukaddes.smsk@gmail.com (M.Ş.)

**Keywords:** bronchiectasis, blood eosinophils, eosinopenia, phenotype, exacerbation, biomarker

## Abstract

*Background and Objectives:* Bronchiectasis exhibits heterogeneous inflammatory features. Although peripheral blood eosinophil counts guide therapy in other airway diseases, their role in phenotyping bronchiectasis remains under-defined. We investigated the clinical, microbiological, and inflammatory profiles of eosinopenic and eosinophilic phenotypes based on stable-state blood eosinophil counts. *Materials and Methods:* This retrospective cohort study evaluated 163 patients diagnosed with bronchiectasis between December 2020 and October 2024. Patients were stratified into three groups based on stable-state peripheral blood eosinophil counts: eosinopenia (≤100 cells/µL), intermediate eosinophil levels (101–299 cells/µL), and eosinophilia (≥300 cells/µL). Baseline demographics, comorbidities, laboratory data, sputum culture results, radiological scores, and clinical outcomes were compared. Factors associated with exacerbations were evaluated using multivariable logistic regression. *Results:* Eosinopenia was present in 30.1% of patients, intermediate eosinophil levels in 46.0%, and eosinophilia in 23.9%. Compared with patients in the intermediate group, those with eosinopenia had significantly higher sputum culture positivity (53.1% vs. 21.3%; *p* < 0.001), more frequent *Pseudomonas aeruginosa* isolation in stable-state sputum cultures (26.5% vs. 9.3%; *p* = 0.011), and a higher neutrophil-to-lymphocyte ratio. Hospitalization rates were numerically higher in the eosinophilia group than in the intermediate group (38.5% vs. 18.7%), although the overall between-group difference did not reach statistical significance (*p* = 0.069). In multivariable analysis, neither eosinopenia nor eosinophilia was significantly associated with exacerbation (adjusted OR 0.88, 95% CI 0.39–2.00; *p* = 0.760 and adjusted OR 1.51, 95% CI 0.56–4.07; *p* = 0.418, respectively). The modified Reiff score showed only a borderline association after adjustment (adjusted OR 1.16, 95% CI 0.99–1.35; *p* = 0.060). *Conclusions:* Blood eosinophil levels were associated with distinct clinical phenotypes in bronchiectasis. Eosinopenia was characterized by higher NLR and more frequent *P. aeruginosa* isolation in stable-state sputum cultures, whereas eosinophilia was associated with a distinct clinical profile. Blood eosinophil counts may serve as a practical phenotyping tool rather than a linear prognostic biomarker.

## 1. Introduction

Bronchiectasis is a complex and heterogeneous chronic airway disease with substantial variability in clinical presentation, disease course, and progression [1]. This heterogeneity reflects differences in underlying etiology, airway inflammatory patterns, microbiological characteristics, and structural lung abnormalities [2]. Exacerbations represent a major component of disease burden and are associated with impaired quality of life, accelerated disease progression, hospitalization, and increased mortality [3]. Accordingly, contemporary bronchiectasis management increasingly emphasizes individualized assessment, including identification of underlying causes, microbiological characteristics, and clinically relevant phenotypes and treatable traits [2].

Although neutrophilic inflammation predominates in bronchiectasis, a distinct subgroup of patients exhibits eosinophilic inflammatory features, with blood eosinophil counts above 300 cells/µL reported in approximately one-fifth of patients without coexisting asthma or other recognized eosinophilic disorders [4]. In contrast to asthma and COPD, however, the clinical utility of blood eosinophil count as a biomarker in bronchiectasis remains uncertain [2]. Available evidence suggesting potential clinical relevance of elevated eosinophil counts is largely derived from observational studies and post-hoc analyses, and current guideline evidence is insufficient to support treatment decisions based solely on blood eosinophil levels [5]. Moreover, the clinical significance of low blood eosinophil counts is even less well characterized. Thus, the associations of both elevated and reduced blood eosinophil levels with clinical, inflammatory, and microbiological characteristics in bronchiectasis remain incompletely understood [2]. Accordingly, this study aimed to compare the clinical, microbiological, and inflammatory characteristics of bronchiectasis patients stratified according to stable-state blood eosinophil counts. By evaluating both eosinopenic and eosinophilic groups within the same cohort, we sought to clarify whether these eosinophil-defined subgroups represent clinically distinct phenotypes and to determine their potential relevance for phenotypic characterization in bronchiectasis.

## 2. Materials and Methods

### 2.1. Study Design

This retrospective cohort study evaluated the association of stable-state peripheral blood eosinophil levels with inflammatory profiles and exacerbations in patients with bronchiectasis. The study protocol was approved by the Institutional Ethics Committee of the University of Health Sciences Sanatorium Training and Research Hospital (24 September 2025/374).

### 2.2. Patient Selection and Data Collection

**Figure 1:**
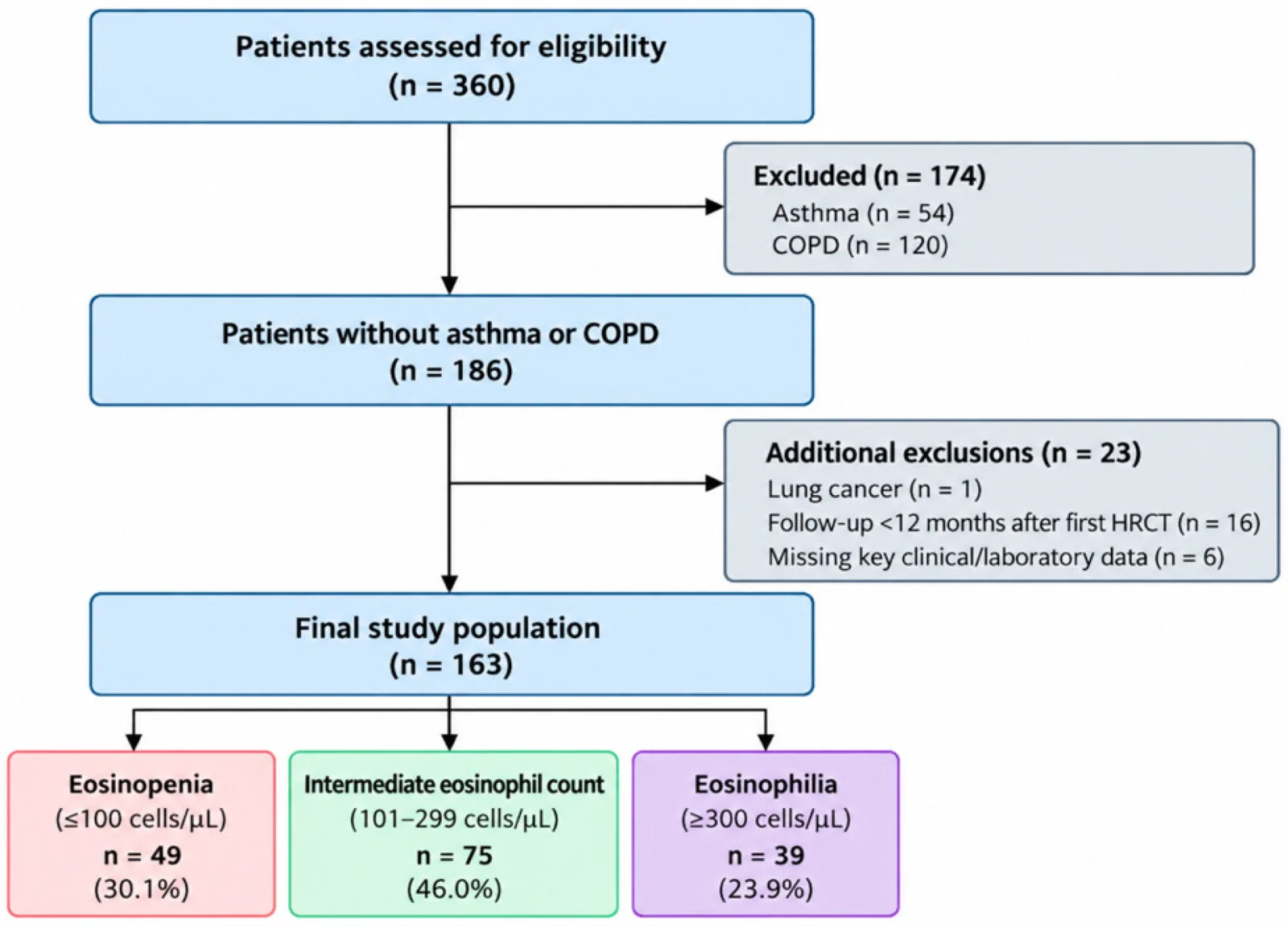
Bronchiectasis patient flowchart.

A total of 360 patients with bronchiectasis who were assessed between December 2020 and October 2024 were screened for eligibility. After excluding 54 patients with asthma and 120 with COPD, 186 patients remained. Of these, 23 were additionally excluded because of lung cancer (*n* = 1), follow-up of <12 months after the first HRCT (*n* = 16), or missing key clinical/laboratory data (*n* = 6), resulting in a final study cohort of 163 patients (Figure 1).

The first HRCT was defined as the index date, and patients were required to have at least 12 months of documented clinical follow-up thereafter. Baseline data included demographics, smoking history, comorbidities, laboratory parameters, HRCT findings, FEV_1_ (% predicted), stable-state maintenance therapies, and sputum microbiology. The blood eosinophil count used for group assignment was obtained on the index HRCT date, provided that the patient was clinically stable, with no documented exacerbation within the four weeks prior to the index date. This baseline measurement preceded the assessment of clinical outcomes during the subsequent 12-month follow-up period. Clinical outcomes, including exacerbation-related outpatient visits, emergency department visits, hospitalizations, intensive care unit (ICU) admissions, and total length of stay, were subsequently assessed during the 12 months following the index date. Exacerbations were retrospectively confirmed by a pulmonologist using standardized criteria, with a second pulmonologist consulted in ambiguous cases. All-cause mortality was recorded through October 2025.

### 2.3. Outcome Definitions

The primary outcomes were differences in clinical, inflammatory, and microbiological characteristics across the predefined blood eosinophil groups. Secondary outcomes included exacerbations and healthcare utilization during the 12-month follow-up, as well as factors associated with exacerbations in multivariable analysis.

### 2.4. Exclusion Criteria

Patients with missing medical or laboratory records, coexisting eosinophilic lung disease, COPD, asthma, allergic bronchopulmonary aspergillosis (ABPA), active malignancy, current systemic corticosteroid use, or less than 1 year of follow-up after the first HRCT were excluded.

Asthma and COPD were identified based on previously established diagnoses documented by a pulmonologist in the medical records and were supported by available pulmonary function test results when present.

### 2.5. Laboratory and Radiological Assessments

Complete blood counts were performed using an automated hematology analyzer (XN-530, Sysmex Corporation, Kobe, Japan). For eosinophil classification, blood eosinophil counts obtained during a clinically stable period were used. Clinical stability was retrospectively verified from medical records and defined as the absence of a documented bronchiectasis exacerbation, emergency department visit or hospitalization for acute respiratory deterioration, and systemic corticosteroid use during the preceding 4 weeks. Blood samples obtained during an acute respiratory event were excluded.

Patients were stratified into three groups according to stable-state peripheral blood eosinophil counts, eosinopenia (≤100 cells/µL), intermediate eosinophil levels (101–299 cells/µL), and eosinophilia (≥300 cells/µL), based on established literature thresholds [6]. The ≤100 cells/µL threshold was retained to preserve adequate subgroup size and statistical power for comparative analyses. Microbiological data were obtained from sputum cultures collected during clinically stable periods. Cultures obtained during documented bronchiectasis exacerbations or acute respiratory deterioration were not used for the microbiological phenotype analysis. Sputum cultures obtained during antibiotic treatment were excluded from the microbiological analysis.

Culture positivity was defined as the isolation of a potentially pathogenic microorganism from a stable-state sputum culture, and *Pseudomonas aeruginosa* isolation was defined as the detection of *P. aeruginosa* in a stable-state sputum culture. Because the retrospective database did not include the number of sputum samples obtained per patient, differences in sampling frequency could not be assessed. Serial sputum cultures were also not systematically available; therefore, chronic bronchial infection could not be reliably determined, and microbiological findings are reported as bacterial isolation rather than chronic infection or colonization. Bronchiectasis severity was assessed using the modified Reiff score (range, 1–18), calculated by multiplying the number of involved lobes (with the lingula considered separately) by the degree of bronchial dilatation (tubular = 1, varicose = 2, cystic = 3) [7]. HRCT images were independently evaluated by two experienced pulmonologists and one radiologist, all blinded to clinical outcomes.

### 2.6. Definitions

Bronchiectasis diagnosis required chronic cough, sputum production, a history of exacerbations, and diagnostic HRCT findings [1]. An exacerbation was defined as a sustained worsening (>48 h) of at least three respiratory symptoms (cough, sputum volume and/or consistency, sputum purulence, breathlessness and/or exercise tolerance, fatigue and/or malaise, or hemoptysis) requiring a change in treatment [3].

### 2.7. Statistical Analysis

Statistical analyses were performed using SPSS version 22.0. Continuous variables are presented as median (interquartile range [IQR]) and categorical variables as frequencies and percentages. Group differences were evaluated using the Mann–Whitney U test, Kruskal–Wallis test, and Pearson’s chi-square or Fisher’s exact test, as appropriate. Significant overall differences among the three groups were further evaluated using post-hoc pairwise comparisons with Bonferroni correction.

Univariable logistic regression analyses were performed to evaluate factors associated with exacerbations. Multivariable logistic regression analysis was performed using age, sex, modified Reiff score, and blood eosinophil category. Covariates were selected on clinical and methodological grounds rather than on univariable statistical significance alone. Individual lobar involvement variables were not included together with the modified Reiff score because the latter incorporates radiological disease extent. To maintain a parsimonious model, smoking status, sputum culture positivity, and NLR were not included in the multivariable analysis. Logistic regression results are reported as odds ratios (ORs) with 95% confidence intervals (CIs). Multicollinearity was assessed using variance inflation factors (VIF) and tolerance values, and model calibration was evaluated using the Hosmer–Lemeshow goodness-of-fit test. FEV_1_ data were available for 76 patients (46.6%). A sensitivity analysis was performed in this subgroup by additionally including FEV_1_ % predicted in the multivariable model. Mortality analysis was not performed because of the low number of events (*n* = 9). No imputation was performed for missing data; analyses were based on available data. Diagnostic performance was evaluated using receiver operating characteristic (ROC) curve analysis. A two-sided *p* value < 0.05 was considered statistically significant.

## 3. Results

The study cohort comprised 163 patients (median age 66 years [IQR 53–72]; 52.1% male). Chronic comorbidities were present in 51.5% of the study population. According to the modified Reiff score, 80.4% had mild radiological disease. Patients were stratified into eosinopenia (30.1%), intermediate eosinophil (46.0%), and eosinophilia (23.9%) groups (Table 1). Sex distribution differed significantly among the eosinophil groups (*p* = 0.039), with a higher proportion of males in the eosinophilia group than in the intermediate group (69.2% vs. 49.3%; post-hoc *p* = 0.042). Stable-state sputum culture positivity differed significantly among the groups (*p* < 0.001) and was more frequent in the eosinopenia group than in the intermediate group (53.1% vs. 21.3%; post-hoc *p* < 0.001). Similarly, stable-state *Pseudomonas aeruginosa* isolation differed significantly among the groups (*p* = 0.038) and was more frequent in the eosinopenia group than in the intermediate group (26.5% vs. 9.3%; post-hoc *p* = 0.011).

Significant between-group differences were also observed in NLR (*p* = 0.001) and lymphocyte counts (*p* < 0.001); compared with the intermediate group, patients with eosinopenia had a higher NLR (post-hoc *p* = 0.003) and lower lymphocyte counts (post-hoc *p* = 0.014). In contrast, no significant differences were observed among the groups in age, comorbidities, smoking status, distribution of affected lung lobes, modified Reiff score and bronchiectasis severity, ICS use, or WBC, neutrophil, PLT, albumin, and CRP levels (all *p* > 0.05) (Table 2).

Regarding healthcare utilization, hospitalization was numerically more frequent in the eosinophilia group than in the intermediate group (38.5% vs. 18.7%); however, the overall difference among the three eosinophil groups did not reach statistical significance (*p* = 0.069). Outpatient visits, emergency department visits, length of hospital stay, and ICU admissions also did not differ significantly among the groups (all *p* > 0.05; Table 3).

In the univariable logistic regression analysis, right lower lobe involvement and the modified Reiff score were significantly associated with exacerbation (OR 0.38, 95% CI 0.18–0.81; *p* = 0.012 and OR 1.17, 95% CI 1.00–1.36; *p* = 0.044, respectively). In the multivariable model including age, sex, modified Reiff score, and blood eosinophil category, neither eosinopenia nor eosinophilia was significantly associated with exacerbation. Compared with the intermediate eosinophil group, the adjusted OR was 0.88 (95% CI 0.39–2.00; *p* = 0.760) for eosinopenia and 1.51 (95% CI 0.56–4.07; *p* = 0.418) for eosinophilia. The association between modified Reiff score and exacerbation did not remain statistically significant after adjustment (adjusted OR 1.16, 95% CI 0.99–1.35; *p* = 0.060) (Table 4). No problematic multicollinearity was detected (VIF range 1.03–1.19), and the Hosmer–Lemeshow test showed no evidence of poor model calibration (*p* = 0.471).

FEV_1_ % predicted was available for 76 patients, including 55 patients with and 21 patients without an exacerbation. In a sensitivity analysis additionally incorporating FEV_1_ % predicted into the multivariable model, the main findings remained unchanged. FEV_1_ was not significantly associated with exacerbation (adjusted OR 1.00 per 1% increase, 95% CI 0.97–1.03; *p* = 0.898), and neither eosinopenia nor eosinophilia showed a statistically significant association with exacerbation (Supplementary Table S1).

**Figure 2:**
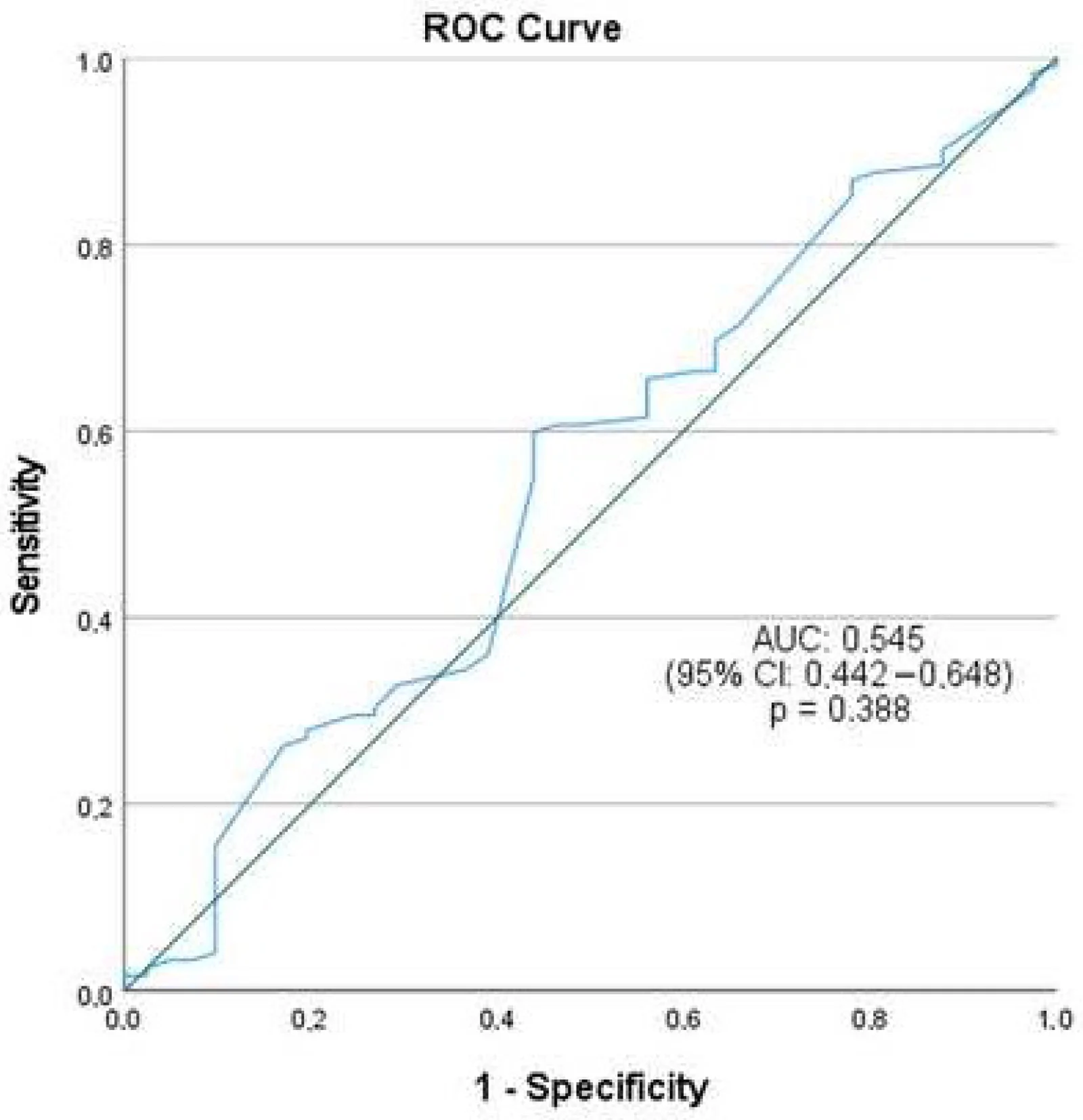
Receiver operating characteristic (ROC) curve for blood eosinophil count as a predictor of exacerbation.The solid curve represents the ROC curve, and the diagonal line represents the reference (no-discrimination) line. AUC = 0.545 (95% CI: 0.442–0.648; *p* = 0.388), indicating that blood eosinophil level alone lacks predictive accuracy for exacerbations.

ROC curve analysis further demonstrated that peripheral blood eosinophil levels had limited discriminatory ability for exacerbations (AUC = 0.545; 95% CI 0.442–0.648; *p* = 0.388) (Figure 2).

## 4. Discussion

Our findings suggest that peripheral blood eosinophil levels in bronchiectasis may identify distinct clinical phenotypes rather than serving as a linear marker of disease severity. Low eosinophil counts (≤100 cells/µL) were associated with an infection-predominant profile characterized by higher NLR, lower lymphocyte counts, and more frequent bacterial, particularly *Pseudomonas aeruginosa*, isolation in stable-state sputum cultures.

Conversely, high eosinophil counts (≥300 cells/µL) were associated with a distinct clinical profile; hospitalization was numerically more frequent in this group, although the overall between-group difference was not statistically significant. Patients with intermediate values (101–299 cells/µL) exhibited heterogeneous features. Taken together, these findings support the use of blood eosinophil levels primarily for phenotypic characterization rather than for direct prediction of disease progression.

These findings are consistent with previous evidence suggesting a non-linear relationship between blood eosinophil counts and bronchiectasis outcomes. Martínez-García et al., in a large multicenter prospective cohort, demonstrated a U-shaped relationship between blood eosinophil counts and both bronchiectasis severity and exacerbations, with a more pronounced association at the eosinopenic end of the spectrum [8]. Our findings extend this observation by suggesting that the opposite extremes of the eosinophil spectrum may be associated with distinct clinical and microbiological characteristics rather than simply reflecting greater disease severity at both ends. This supports the potential role of blood eosinophil counts as a phenotyping tool in bronchiectasis. Although bronchiectasis is classically regarded as a predominantly neutrophilic inflammatory disease, recent studies have identified eosinophil-associated clinical phenotypes [6]. Proposed mechanisms underlying eosinophilic inflammation include type 2 immune pathways involving IL-5 and IL-13 [9,10], whereas eosinophils also contribute to antibacterial host defense through phagocytosis, granule proteins, and extracellular DNA traps [4,11]. Accordingly, reduced eosinophil levels have been proposed to be associated with infectious outcomes through alterations in host defense.

Associations between low eosinophil levels and adverse clinical or infectious outcomes have also been reported in previous studies. Izhakian et al. found that eosinopenia (<100 cells/µL) was associated with a poorer clinical course, more frequent hospitalizations, and increased mortality risk [11]. Similarly, in patients with COPD, low peripheral blood eosinophil levels have been linked to bacterial colonization [12]. More specifically in bronchiectasis, Zhang et al. reported a higher frequency of *Pseudomonas aeruginosa* isolation among patients with eosinopenia (<100 cells/µL) [13]. In our cohort, eosinopenia was likewise associated with more frequent bacterial and *P. aeruginosa* isolation in stable-state sputum cultures, together with higher NLR and lower lymphocyte counts. These findings suggest that marked eosinopenia may be associated with an infection-predominant clinical profile and may identify patients in whom closer microbiological surveillance could be warranted. By contrast, eosinophilia at the opposite end of the eosinophil spectrum has also been associated with distinct clinical characteristics in bronchiectasis.

Prospective cohorts indicate that eosinophilic patients experience more frequent and severe exacerbations [8,14]. In our study, hospitalization was numerically more frequent in the eosinophilia group, but the overall between-group comparison did not reach statistical significance. Therefore, our findings do not establish an independent association between eosinophilia and hospitalization, although they remain compatible with the concept of blood eosinophils as a potential “treatable trait” in bronchiectasis [15]. Consistent with this concept, the current ERS guideline emphasizes individualized assessment of underlying etiology, microbiological characteristics, inflammatory phenotypes, and comorbidities to identify clinically relevant treatable traits [2]. Within this framework, blood eosinophil counts have attracted particular interest as a potential biomarker of treatment response, especially in relation to inhaled corticosteroid (ICS) therapy. In analyses from the EMBARC cohort stratified by ICS use, no significant differences were observed among blood eosinophil groups in exacerbation frequency or overall disease severity [16]. Similarly, in our cohort, the proportion of patients receiving ICS did not differ significantly across eosinophil groups. In contrast, Martínez-García et al. reported that ICS use in patients with an eosinophilic phenotype was associated with a lower exacerbation frequency [8]. Although these findings suggest a potential treatment-response signal in a subgroup with elevated eosinophil counts, the current ERS guideline emphasizes that the available evidence remains insufficient to recommend ICS therapy in bronchiectasis based solely on blood eosinophil levels [2]. This potential, but as yet unconfirmed, treatment-response signal has also prompted interest in more targeted therapies directed against eosinophilic inflammation, particularly biologics targeting the IL-5/IL-5R pathway. However, evidence regarding their clinical efficacy remains limited and inconsistent. The phase 3 MAHALE trial of benralizumab demonstrated robust eosinophil suppression but failed to show a statistically significant reduction in annual exacerbation rates or improvement in quality of life compared with placebo, potentially due to the small sample size, underrepresentation of the target population (≥300 cells/µL), and baseline imbalances [17]. In contrast, smaller real-world series and pilot studies evaluating mepolizumab or benralizumab in patients with severe asthma and concomitant bronchiectasis reported marked clinical and functional benefits [18,19,20]. These discrepancies highlight the need for large-scale prospective trials to clarify the clinical role of biologic therapies in eosinophilic bronchiectasis.

The lack of a significant association between blood eosinophil categories and exacerbation in our cohort suggests that BEC may be more useful for phenotypic characterization than for direct risk prediction. Previous multicohort data similarly indicate that blood eosinophil levels are associated with distinct clinical and microbiological characteristics, whereas their relationship with exacerbation risk is not consistently linear across populations. Taken together, these findings suggest that the potential clinical value of BEC in bronchiectasis may lie primarily in distinguishing clinically relevant phenotypes rather than in independently predicting exacerbation risk. This study has several limitations. Its retrospective, single-center design and modest sample size may limit generalizability. Pulmonary function data were available for only 46.6% of patients; therefore, unrecognized obstructive airway disease, particularly in the eosinophilia group, cannot be entirely excluded. Pre-index exacerbation history and annual exacerbation frequency were not systematically available. Consequently, multidimensional severity scores such as BSI and FACED could not be reliably calculated for the full cohort. Serial sputum cultures and per-patient sampling frequency were also unavailable; thus, microbiological findings should be interpreted as stable-state bacterial isolation rather than chronic infection or colonization. Eosinophil classification was based on a single stable-state measurement, and comprehensive etiological classification was unavailable, leaving the possibility of residual confounding. Long-term macrolide and inhaled antibiotic use could not be reliably assessed as separate treatment categories. The higher proportion of male patients in the eosinophilia group may also represent a source of between-group imbalance. Finally, given the high prevalence of exacerbations, odds ratios should not be interpreted as direct estimates of relative risk. Nevertheless, exclusion of patients with asthma, COPD, ABPA, other eosinophilic lung diseases, or current systemic corticosteroid use reduced potential confounding of blood eosinophil levels.

## 5. Conclusions

In conclusion, blood eosinophil levels in bronchiectasis were associated with distinct clinical phenotypes. Eosinopenia was characterized by higher NLR and more frequent *P. aeruginosa* isolation in stable-state sputum cultures, whereas eosinophilia was associated with a distinct clinical profile. Peripheral blood eosinophil counts may provide a practical and accessible tool for phenotypic stratification in bronchiectasis; however, their clinical relevance and temporal stability should be confirmed in prospective studies incorporating repeated eosinophil measurements.

## Figures and Tables

**Table 1:** General characteristics of patients with bronchiectasis.

Characteristic	*n* = 163, Median (Q1–Q3) or *n* (%)
Demographic Characteristics	
Age (years)	66 (53–72)
Sex, *n* (%)	Male: 85 (52.1); Female: 78 (47.9)
Comorbidities	
Any comorbidity	84 (51.5)
Hypertension (HT)	61 (37.4)
Coronary artery disease (CAD)	38 (23.3)
Diabetes mellitus (DM)	36 (22.1)
Previous tuberculosis (TB)	20 (12.3)
Smoking Status	
Current smoker	42 (25.8)
Never smoker	65 (39.9)
Ex-smoker	56 (34.4)
Radiology	
Number of affected lobes	2 (1–3)
Modified Reiff score	4 (2–6)
Bronchiectasis severity, *n* (%)	Mild: 131 (80.4); Moderate: 27 (16.6); Severe: 5 (3.1)
Blood Eosinophil Count Group	
Eosinopenia (≤100 cells/µL)	49 (30.1)
Intermediate Eosinophil Group (101–299 cells/µL)	75 (46.0)
Eosinophilia (≥300 cells/µL)	39 (23.9)
Clinical Outcomes	
Exacerbation during follow-up	122 (74.8)
All-cause mortality	9 (5.5)
Sputum Culture	
Positive culture	53 (32.5)
*Pseudomonas aeruginosa*	26 (16.0)
*Streptococcus pneumoniae*	26 (16.0)
*Haemophilus influenzae*	17 (10.4)
Laboratory Parameters	
Eosinophil count (cells/µL)	200 (100–290)
White blood cell count (WBC, ×10^3^/µL)	8400 (6700–11,000)
Neutrophil-to-lymphocyte ratio (NLR)	2.5 (1.6–4.2)
C-reactive protein (CRP, mg/L)	3.3 (1.0–12.2)
Albumin (g/L)	40.5 (38.0–43.0)

Continuous variables are presented as median (Q1–Q3); categorical variables as number (%). Bronchiectasis severity was assessed using the modified Reiff score.

**Table 2:** Key clinical, microbiological, and inflammatory characteristics according to blood eosinophil count.

Variable	Intermediate Eosinophil Group (*n* = 75)	Eosinopenia (*n* = 49)	Eosinophilia (*n* = 39)	Global *p*	Significant Pairwise Comparison *
Male sex, *n* (%)	37 (49.3)	21 (42.9)	27 (69.2)	**0.039**	Intermediate eosinophil vs. eosinophilia, *p* = 0.042
Modified Reiff score	4 (2–6)	5 (3–6)	4 (2–7)	0.444	—
Positive sputum culture, *n* (%)	16 (21.3)	26 (53.1)	11 (28.2)	**<0.001**	Intermediate eosinophil vs. eosinopenia, *p* < 0.001
*Pseudomonas aeruginosa*, *n* (%)	7 (9.3)	13 (26.5)	6 (15.4)	**0.038**	Intermediate eosinophil vs. eosinopenia, *p* = 0.011
NLR	2.4 (1.6–3.7)	3.9 (2.0–6.9)	2.3 (1.6–3.1)	**0.001**	Intermediate eosinophil vs. eosinopenia, *p* = 0.003
Lymphocyte (/µL)	1980 (1700–2570)	1810 (1110–2240)	2400 (1900–3000)	**<0.001**	Intermediate eosinophil vs. eosinopenia, *p* = 0.014
ICS use, *n* (%)	10 (13.3)	3 (6.1)	3 (7.7)	0.368	—

Continuous variables are presented as median (Q1–Q3) and categorical variables as *n* (%). NLR: neutrophil-to-lymphocyte ratio; ICS: inhaled corticosteroid. Bold values indicate statistical significance (*p* < 0.05). * Only statistically significant pairwise comparisons are shown.

**Table 3:** Comparison of exacerbation status, healthcare utilization, and hospitalization characteristics by blood eosinophil count group.

Variable	Intermediate Eosinophil Group (*n* = 75)	Eosinopenia (*n* = 49)	Eosinophilia (*n* = 39)	*p* Value
Exacerbation Status				
Exacerbation during follow-up	55 (73.3)	35 (71.4)	32 (82.1)	0.479
Outpatient Clinic Visits				
Number of outpatient visits	2 (1–3)	2 (1.5–3.0)	2 (1–3)	0.437
<2 visits	21 (28.0)	17 (34.7)	9 (23.1)	0.478
≥2 visits	54 (72.0)	32 (65.3)	30 (76.9)	
Emergency Department Visits				
<2 visits	68 (90.7)	44 (89.8)	37 (94.9)	0.667
≥2 visits	7 (9.3)	5 (10.2)	2 (5.1)	
Hospitalization				
None	61 (81.3)	37 (75.5)	24 (61.5)	0.069
≥1	14 (18.7)	12 (24.5)	15 (38.5)	
Length of hospital stay (days)	7 (5.8–9.0)	12 (5.2–14.8)	8 (6–12)	0.395
Intensive Care Unit Admission				
No ICU admission	73 (97.3)	47 (95.9)	38 (97.4)	0.885
1 ICU admission	2 (2.7)	2 (4.1)	1 (2.6)	

Continuous variables are presented as median (Q1–Q3); categorical variables as *n* (%). ICU: intensive care unit. The *p* value represents the overall comparison among the three eosinophil groups.

**Table 4:** Univariable and multivariable logistic regression analyses of factors associated with exacerbations in patients with bronchiectasis.

Variable	Univariable OR (95% CI)	*p* Value	Multivariable OR (95% CI)	*p* Value
Age, per year	0.99 (0.97–1.02)	0.584	0.99 (0.96–1.02)	0.540
Sex				
Male	Reference	—	Reference	—
Female	0.64 (0.32–1.31)	0.224	0.73 (0.35–1.55)	0.418
Smoking status				
Never smoker	Reference	—	—	—
Current smoker	1.89 (0.74–4.80)	0.182	—	—
Former smoker	1.47 (0.65–3.32)	0.353	—	—
Right lower lobe involvement	0.38 (0.18–0.81)	0.012	—	—
Left lower lobe involvement	0.73 (0.35–1.50)	0.392	—	—
Modified Reiff score, per point	1.17 (1.00–1.36)	0.044	1.16 (0.99–1.35)	0.060
Positive sputum culture	2.01 (0.88–4.58)	0.099	—	—
NLR, per unit	1.07 (0.95–1.21)	0.258	—	—
Blood eosinophil category				
Intermediate BEC (101–299 cells/µL)	Reference	—	Reference	—
Eosinopenia (≤100 cells/µL)	0.91 (0.41–2.03)	0.816	0.88 (0.39–2.00)	0.760
Eosinophilia (≥300 cells/µL)	1.66 (0.63–4.36)	0.302	1.51 (0.56–4.07)	0.418

Note. The multivariable model included age, sex, modified Reiff score, and blood eosinophil category. Smoking status, individual lobar involvement, sputum culture positivity, and NLR were evaluated only in univariable analyses. OR, odds ratio; CI, confidence interval; BEC, blood eosinophil count; NLR, neutrophil-to-lymphocyte ratio.

## Data Availability

The data presented in this study are available on request from the corresponding author. The data are not publicly available due to privacy and ethical restrictions on patient data.

## Supplementary Materials

The following supporting information can be downloaded at https://www.mdpi.com/article/10.3390/medicina62101982/s1: Table S1: Sensitivity analysis of factors associated with exacerbation in patients with available FEV_1_ data (*n* = 76).

## References

[1] Aliberti S., Goeminne P.C., O’Donnell A.E., Aksamit T.R., Al-Jahdali H., Barker A.F., Blasi F., Boersma W.G., Crichton M.L., De Soyza A. (2022). Criteria and definitions for the radiological and clinical diagnosis of bronchiectasis in adults for use in clinical trials: International consensus recommendations. Lancet Respir. Med..

[2] Chalmers J.D., Haworth C.S., Flume P., Long M.B., Burgel P.R., Dimakou K., Blasi F., Herrero-Cortina B., Dhar R., Chotirmall S.H. (2025). European Respiratory Society clinical practice guideline for the management of adult bronchiectasis. Eur. Respir. J..

[3] Hill A.T., Haworth C.S., Aliberti S., Barker A., Blasi F., Boersma W., Chalmers J.D., De Soyza A., Dimakou K., Elborn J.S. (2017). Pulmonary exacerbation in adults with bronchiectasis: A consensus definition for clinical research. Eur. Respir. J..

[4] Shoemark A., Shteinberg M., De Soyza A., Haworth C.S., Richardson H., Gao Y., Perea L., Dicker A.J., Goeminne P.C., Cant E. (2022). Characterization of eosinophilic bronchiectasis: A European multicohort study. Am. J. Respir. Crit. Care Med..

[5] Aliberti S., Sotgiu G., Blasi F., Saderi L., Posadas T., Martinez Garcia M.A. (2020). Blood eosinophils predict inhaled fluticasone response in bronchiectasis. Eur. Respir. J..

[6] Martins M., Keir H., Chalmers J. (2023). Endotypes in bronchiectasis: Moving towards precision medicine. A narrative review. Pulmonology.

[7] Reiff D.B., Wells A.U., Carr D.H., Cole P.J., Hansell D.M. (1995). CT findings in bronchiectasis: Limited value in distinguishing between idiopathic and specific types. AJR Am. J. Roentgenol..

[8] Martínez-García M.Á., Méndez R., Olveira C., Girón R., García-Clemente M., Máiz L., Sibila O., Golpe R., Rodríguez-Hermosa J.L., Barreiro E. (2023). The U-shaped relationship between eosinophil count and bronchiectasis severity: The effect of inhaled corticosteroids. Chest.

[9] Fouka E., Lindén A., Bossios A. (2025). The role of T-helper and T regulatory cells in driving neutrophilic and eosinophilic inflammation in bronchiectasis. Front. Immunol..

[10] Chalmers J., Elborn S., Greene C. (2023). Basic, translational and clinical aspects of bronchiectasis in adults. Eur. Respir. Rev..

[11] Izhakian S., Yehezkely R., Frajman A., Mekiten O., Hadar O., Rockland A., Malka L., Freidkin L., Rosengarten D., Kramer M.R. (2025). Eosinopenia in bronchiectasis: A novel biomarker for morbidity and mortality. Chron. Respir. Dis..

[12] Wang F., Yang L., Wang M., He B. (2022). Association between blood eosinophil count and bacterial infection and clinical outcomes in patients with severe exacerbations of chronic obstructive pulmonary disease. Chin. Med. J..

[13] Zhang R.Y., Liu X.F., Chen Q.L., Yao X.J., Wang Y.H., Li R., Wang X.M., He Y.Y., Zhang X.C. (2026). Blood eosinophil count and clinical characteristics in patients with bronchiectasis. Zhonghua Jie He He Hu Xi Za Zhi.

[14] Campisi R., Nolasco S., Mancuso M., Spinella M., Vignera F., Crimi N., Vancheri C., Crimi C. (2024). Eosinophilic bronchiectasis: Prevalence, severity, and associated features—A cohort study. J. Clin. Med..

[15] Pollock J., Goeminne P. (2023). Eosinophils in bronchiectasis: A U-turn for bronchiectasis management. Chest.

[16] Pollock J., Huang J.T., Shuttleworth M., Long M.B., Richardson H., Alferes de Lima D., Kuzmanova E., Clarke C., Shteinberg M., Aliberti S. (2026). Clinical, molecular and microbial characterisation of the eosinophilic endotype of bronchiectasis (EMBARC-BRIDGE). Thorax.

[17] Chalmers J.D., Aliberti S., Burr L., Weinreich U.M., Bergna M.Á., Åstrand A., Basavanna P., Fan Y., Gatti M., Ho C.N. (2026). Benralizumab in bronchiectasis: Results from the phase 3 MAHALE study. ERJ Open Res..

[18] Oriano M., Gramegna A., Amati F., D’Adda A., Gaffuri M., Contoli M., Bindo F., Simonetta E., Di Francesco C., Santambrogio M. (2021). T2-high endotype and response to biological treatments in patients with bronchiectasis. Biomedicines.

[19] Crimi C., Campisi R., Nolasco S., Cacopardo G., Intravaia R., Porto M., Impellizzeri P., Pelaia C., Crimi N. (2021). Mepolizumab effectiveness in patients with severe eosinophilic asthma and co-presence of bronchiectasis: A real-world retrospective pilot study. Respir. Med..

[20] Rademacher J., Konwert S., Fuge J., Dettmer S., Welte T., Ringshausen F.C. (2020). Anti-IL5 and anti-IL5Ra therapy for clinically significant bronchiectasis with eosinophilic endotype: A case series. Eur. Respir. J..

