# Supplementary material for: Beyond Eosinophilia: A Dual Eosinophil Phenotype Model in Bronchiectasis
Source: Medicina (Kaunas). 2026 Oct 14;62(10):1982. doi: 10.3390/medicina62101982 (PMC13707330; doi:10.3390/medicina62101982)
Supplement: Supplementary file 1 [file medicina-62-01982-s001.zip › medicina-4606235-supplementary.pdf]

**Supplementary Table S1. Sensitivity analysis of factors associated with exacerbation in patients with available $FEV_1$ data (n=76).**

| Predictor | Adjusted OR (95% CI) | p value |
|---|---|---|
| Age, per year | 1.01 (0.96–1.06) | 0.716 |
| Female vs male | 0.41 (0.13–1.37) | 0.149 |
| Modified Reiff score, per point | 1.10 (0.88–1.36) | 0.405 |
| BEC ≤100 vs 101–299 cells/μL | 0.89 (0.24–3.30) | 0.861 |
| BEC ≥300 vs 101–299 cells/μL | 2.17 (0.50–9.41) | 0.302 |
| $FEV_1$ % predicted, per 1% increase | 1.00 (0.97–1.03) | 0.898 |

**Note.** The analysis included 76 patients, of whom 55 experienced an exacerbation and 21 did not. Male sex and BEC 101–299 cells/μL were used as reference categories. OR, odds ratio; CI, confidence interval; BEC, blood eosinophil count; $FEV_1$, forced expiratory volume in 1 second.
